# Eugenol as a Promising Molecule for the Treatment of Dermatitis: Antioxidant and Anti-inflammatory Activities and Its Nanoformulation

**DOI:** 10.1155/2018/8194849

**Published:** 2018-12-11

**Authors:** Amanda de Araújo Lopes, Francisco Noé da Fonseca, Talita Magalhães Rocha, Lyara Barbosa de Freitas, Emmanuel Vinicius Oliveira Araújo, Deysi Viviana Tenazoa Wong, Roberto César Pereira Lima Júnior, Luzia Kalyne Almeida Moreira Leal

**Affiliations:** ^1^Pharmaceutical and Cosmetics Studies Center, Faculty of Pharmacy, Dentistry and Nursing, Federal University of Ceará, Fortaleza, Ceará, Brazil; ^2^Department of Physiology and Pharmacology, Federal University of Ceará, Fortaleza, Ceará, Brazil

## Abstract

Contact dermatitis produces an inflammatory reaction primarily via stimulation of keratinocytes and cells of the immune system, which promote the release of cytokines, reactive oxygen species (ROS), and other chemical mediators. Eugenol (EUG, phenylpropanoid of essential oils) has attracted attention due to its anti-inflammatory properties, as well as antioxidant effect. On the other hand, it is volatile and insoluble and is a skin irritant. In this case, nanostructured systems have been successfully employed as a drug carrier for skin diseases since they improve both biological and pharmaceutical properties of active compounds. The cytotoxic, antioxidant, and anti-inflammatory effects of EUG were assessed in human neutrophils and keratinocytes. Additionally, polymeric nanocarries (NCEUG) were prepared to improve the chemical and irritant characteristics of EUG. EUG presented apparent safety and antioxidant and anti-inflammatory effects on human neutrophils, but presented cytotoxic effects on keratinocytes. However, the nanocapsules were able to reduce its cytotoxicity. An *in vivo* experiment of irritant contact dermatitis (ICD) in mice induced by TPA showed that NCEUG reduced significantly the ear edema in mice when compared to the EUG solution, as well as the leukocyte infiltration and IL-6 level, possibly due to better skin permeation and irritancy blockage. These findings suggest that EUG is a promising bioactive molecule, and its nanoencapsulation seems to be an interesting approach for the treatment of ICD.

## 1. Introduction

Contact dermatitis accounts for 70–90% of all occupational skin diseases and describes the allergic skin reaction resulting from exposure to irritants (irritant contact dermatitis (ICD)) or to allergens (allergic contact dermatitis (ACD)) [1].

Several alterations are observed in epidermal cells during ICD. In some cases, such changes are limited to the superficial layers of the epidermis. In the course of the inflammatory process, the production of IL-8 by keratinocytes with consequent recruitment of polymorphonuclear neutrophils (PMNs) accumulates in the damaged epidermis, leading to the formation of intraepidermal pustules [2].

In ICD, an array of reactive oxygen species (ROS) is produced. ROS are set free by inflammatory mediators, generated directly by irritants [3–5], and released during free radical chain reactions. The main source of ROS is inflammatory cellular infiltrate. Stimulated cells produce superoxide; the respiratory burst of infiltrating PMNs in inflamed skin will produce high local levels of superoxide anion and hydrogen peroxide. Excessive production of ROS results in peroxidation of cell membrane lipids and damages of proteins and DNA [6, 7]. The ROS such as superoxide can rapidly combine with nitric oxide (NO) to form reactive nitrogen species (RNS), such as peroxynitrite, and is three to four times faster than the dismutation of superoxide by superoxide dismutase (SOD). The RNS, in turn, induces nitrosative stress, which adds to the proinflammatory burden of ROS in keratinocytes [8].

Despite the enormous progress made in recent decades regarding the understanding of the pathophysiology of contact dermatitis, the ideal pharmacotherapy of both irritating as allergic contact dermatitis remains a research target [9]. Despite the existence of anti-inflammatory drugs such as corticosteroids, the topical therapies have disadvantages, such as the induction of cell atrophy, decreased skin barrier regeneration, and alteration of the dermal barrier. In this case, the search for new options for the treatment of ICD becomes necessary.

Eugenol (4-allyl-2-methoxyphenol (EUG)) is a terpene and presents itself as yellow viscous oil at normal temperature. Previous studies have reported biological activities of EUG including antibacterial [10], antifungal [11, 12], and antiallergic [13, 14] properties. The antiasthmatic effect of EUG has been recently reported in a mouse model where EUG was shown to influence the vitamin D3-upregulated protein 1/NF-*κ*B pathway [15]. Also, the inhibition effect of EUG on key enzymes related to diabetes and hypertension [16] was reported in a study that involved both in vitro and *in vivo* model systems. Furthermore, it has been shown that EUG suppresses the activity of Cl^−^ Channel TMEM16A [17]. Recently, EUG has attracted the attention of many researchers because of its anti-inflammatory and chemoprotective effects as well as its antioxidant activity [18] due to the phenolic group present in its structure. Because of wide pharmacological and biological activities, studies with the EUG and plant species that contain this substance are still a priority in research. With regard to the inflammatory process, several studies demonstrate the anti-inflammatory activity of EUG. It was shown that EUG promoted a reduction in carrageenan-induced pleural volumes in mice [19]. Furthermore, EUG causes inhibition of proinflammatory mediators such as COX-2, NF-*κ*B, IL-6, leukotriene C4, and 5-LOX [20–22].

Nanoparticles are colloidal structures (~200 nm) generally made of polymers or lipids which have been used as a drug delivery system since they have the ability to effectively deliver drugs to target sites. Specifically, nanocapsules are a reservoir system composed of an oily core surrounded by a polymer membrane and they are of interest for skin administration because of controlled release of encapsulated active ingredients, which need to diffuse through the polymeric matrix to permeate the skin. Their small size ensures a close interaction with the stratum corneum, and due to their occlusive properties (film formation on the skin), an increased skin hydration effect can be achieved [23, 24]. Also, the penetration of the active compound in the skin layers can be modulated depending on the nanoparticle surface charge; since the biological membranes are negatively charged, anionic nanocarriers have a low penetration profile as cationic ones can reach the deep dermis [25].

Despite the potential pharmacological properties of EUG, it is volatile and presents an irritant effect on the skin, resulting in allergic reactions which limit its use in topic formulations [26]. To overcome it and explore the pharmacological potential of EUG, the present research is aimed at investigating the cytotoxicity and anti-inflammatory profile of EUG both in vitro and *in vivo*, as well as to develop a nanoformulation for topical application. As strategy, polymeric nanocapsules constituted of Eudragit® S100 (an anionic copolymer composed of methacrylic acid and methyl methacrylate) were prepared to retain its penetration in the superficial layers of the skin, since the negative charge of the polymer reduces its interaction with skin cells and the drug penetration [27], which is desirable for dermatitis.

## 2. Material and Methods

### 2.1. Chemicals

Eugenol (EUG), dexamethasone, medium-chain triglycerides (Miglyol®), 12-O-tetradecanoylphorbol-13-acetate (TPA), dimethyl sulfoxide (DMSO), bromide, 3[4,5-dimethylthiazol-2-yl]-2,5-diphenyltetrazolium bromide (MTT), Triton X-100, 3,3′,3,5′-tetramethylbenzidine (TMB), hexadecyltrimethylammonium bromide (HTAB) solution, polysorbate 80, lucigenin (*N,N*′-dimethyl-9,9′-biacridinium dinitrate), luminol (5-amino-2,3-dihydro-1,4-phthalazinedione), Trypan Blue, and quercetin were purchased from Sigma-Aldrich (St. Louis, MO, USA). Eudragit® S100 was obtained from Evonik (Essen, Germany). DMEM culture medium, gelatin (microbiological grade), and fetal bovine serum were purchased from Difco, Becton, Dickinson and Company in Sparks, Detroit, MN, USA.

### 2.2. Isolation of Human Neutrophils

Human leukocyte-rich blood from healthy adults was obtained from HEMOCE (blood bank), Fortaleza, Brazil. Neutrophils were isolated by Lucisano and Mantovani's method [28] with slight modifications [29]. In the present study, the cell suspension contained 80–90% neutrophils with viability of 90 ± 2.0% established by the exclusion with Trypan Blue.

### 2.3. Human Keratinocytes

Human keratinocyte cells (HaCaT) were obtained from the Rio de Janeiro Cell Bank, Brazil. They were routinely grown in 150 cm^2^ tissue culture flasks in DMEM, supplemented with 1% (*v*/*v*) of an antibiotic solution containing 5 mg of penicillin, 5 mg of streptomycin, and 10 mg of neomycin per mL and 7.5% or 10.0% (*v*/*v*) heat-inactivated fetal bovine serum at 37°C under 5% CO_2_.

### 2.4. Animals

The experiments were conducted using male albino mice (*Mus musculus*) of Swiss variety (25 to 30 g), from the Animal House of the Federal University of Ceará. The animals were maintained at 22 ± 2°C, 12/12 h light/dark cycle, and provided food and water *ad libitum*. Experiments were performed according to the Guide of Care and Use of Laboratory Animals from the European Community. All the experimental protocols were previously approved by our Institutional Ethics Committee (#62/16).

### 2.5. Preclinical In Vitro Evaluation of EUG: Cytotoxicity and Anti-inflammatory and Antioxidant Properties

#### 2.5.1. MTT Test

In a preliminary study, human neutrophils (2.5 × 10^6^ cells/mL) were incubated for 30 minutes at 37°C in the presence of EUG (5, 10, 25, 50, and 100 *μ*g/mL) or DMSO (1%, vehicle control), Hank's balanced salt solution (HBSS: nontreated cells), and Triton X-100 (0.2%—standard cytotoxic) in a 96-well plate. After this period, the plate was centrifuged at 2000 rpm for 15 minutes at 25°C and the supernatant discarded as well as a new incubated solution (200 *μ*L) containing 10% of MTT at a concentration of 10 mg/mL, and these cells were incubated again for another 3 hours. Finally, the plate was centrifuged again under the same conditions as above; the supernatant was discarded and then 150 *μ*L of pure DMSO added for cell lysis and solubilization of formazan. At this time, the plates were shaken for 15 minutes. The absorbance was measured by a microplate reader at 550 nm. Cell viability was expressed by percentage [30]. In addition, taking into account that the skin is composed of specific cell types and EUG could be used as treatment for ICD, further MTT tests were conducted using human keratinocytes. For that, the cells were incubated with EUG (same doses), but at different times (24, 48, or 72 h) in order to observe the effect of both short- and long-term exposure.

#### 2.5.2. Cell Membrane Integrity

Cell membrane integrity was assessed by flow cytometry according to the method described by [31]. Neutrophils (2.5 × 10^6^ cells/mL) were incubated with EUG (10, 25, and 50 *μ*g/mL) or DMSO (vehicle control—1%) for 30 minutes. Subsequently, cell pellets were suspended in 300 *μ*L of a hypotonic solution of PI (2 *μ*g/mL) and sodium citrate (0.1%). Under these conditions, the cells were incubated for 45 minutes at room temperature and in the dark. In this assay, cells with membrane disrupted the entry of propidium iodide and emitted high fluorescence. Fluorescence was measured in the FL2 site (orange-red fluorescence—585/42 nm) by flow cytometry (FACSCalibur, Benton Dickinson, CA, USA). Ten thousand events were acquired per sample.

#### 2.5.3. Degranulation Assay

Following Boyum [32], neutrophils (2.5 × 10^6^ cells/mL) were suspended in buffered HBSS. The cells were incubated with EUG (5, 10, 25, 50, and 100 *μ*g/mL), indomethacin (100 *μ*M, standard drug), DMSO (1% *v*/*v*, vehicle), or HBSS (not treated cells group) for 15 min at 37°C. Human neutrophils were stimulated by the addition of PMA (0.1 *μ*M) for 15 min at 37°C. The reaction was stopped by cooling, and the suspension was centrifuged at 2000 g for 10 min at 4°C. Aliquots (50 *μ*L) of the supernatants were added to phosphate-buffered saline [PBS (100 *μ*L)], phosphate buffer (50 *μ*L, pH 7.0), and H_2_O_2_ (0.012%). After 5 min at 37°C, 3,3′,5,5′-tetramethylbenzidine (1.5 mM, 20 *μ*L) was added, and the reaction was stopped with 30 *μ*L of sodium acetate (1.5 M, pH 3.0). The results are expressed as percentage of inhibition of the release of MPO by stimulated human neutrophils.

#### 2.5.4. ROS Production by Human Neutrophils

The chemiluminescent probes—luminol (280 *μ*M) or lucigenin (150 *μ*M)—and EUG (1, 10, 50 and 100 *μ*g/mL), DMSO (1%, vehicle control), and quercetin (25 *μ*g/mL, reference compound) were added to neutrophil suspensions (2.5 × 10^6^ cells/mL) and incubated for 30 min at 37°C. The reaction was initiated by adding PMA (0.1 *μ*M). Chemiluminescence (CL) responses were measured in a luminometer (Synergy HT, BioTek Instruments), where light emission was recorded in c.p.m. (counted photons per min) for 20 min at 37°C. Background CL, produced by nonstimulated cells, was also determined. Results were expressed by the emission of chemiluminescence [33].

### 2.6. Nanoformulation of EUG: Development and Preclinical In Vitro Evaluation

#### 2.6.1. Preparation and Physical Chemical Characterization

Nanocapsule suspensions were prepared by interfacial deposition of the preformed polymer, where an organic phase containing the polymer and oil is poured into an aqueous phase containing the surfactant [34]. Considering that EUG is a volatile oil and it was lost during the concentration procedure in a preliminary study, the formulation composition and preparation was adapted as described elsewhere [35]. So, the organic phase consisted of the anionic methacrylate polymer Eudragit® S100 (0.5 g) and EUG (0.25 g) dissolved in acetone (15 mL) and the aqueous phase (23 mL) contained polysorbate 80 as surfactant (0.192 g). Blank formulations (NCB) were prepared omitting EUG in the organic phase and replacing it for medium-chain triglycerides (Miglyol®, 160 *μ*L). All formulations were prepared in triplicate. Particle sizes and polydispersity indices (*n* = 3) were measured by photon correlation spectroscopy (Zetasizer Nano ZS, Malvern, UK). The samples were diluted 1 : 100 (*v*/*v*) in ultrapure water, and the measurements were performed in triplicate. The zeta potential values were determined by electrophoretic mobility using the same instrument at 25°C and after the sample dilution (1 : 100, *v*/*v*) in 10 mM NaCl. The pH values of the formulations were determined using a calibrated potentiometer (Hanna, Rhode Island, USA), at room temperature. The EUG content in the nanocapsule suspension was performed using a chromatographic system (Waters, USA) coupled with a diode array detector (HPLC-PDA), autosampler, and column oven. The separation was carried out using a C18 column (Phenomenex, 250 × 4.6 mm, 5 *μ*m) coupled to a precolumn (Phenomenex) with similar constitution at 40°C. The mobile phase consisted of water : methanol (85: 15, *v*/*v*), and it was eluted in isocratic mode (0.8 mL/min). The detection was set at 280 nm [36]. Encapsulation efficiency was performed by ultrafiltration-centrifugation as described elsewhere [37]. Additionally, a preliminary stability study was carried out. Formulations were packaged in amber glass containers and stored at room temperature (25 ± 2°C) and protected from light. They were monitored during 2 months by means of size, polydispersity, zeta potential, and pH. Also, the EUG content was determined after 30 and 60 days of storage.

#### 2.6.2. Cytotoxicity Evaluation

In order to evaluate the safety of the nanocarriers (containing or not EUG) for the treatment of ICD, the MTT test was performed using human keratinocytes as previously described. The cells were incubated with NCEUG (5, 10, 25, 50, and 100 *μ*g/mL) or NCB (volume correspondent to the higher volume of NCEUG), Hank's balanced salt solution (HBSS: nontreated cells), or Triton X-100 (0.2%—standard cytotoxic) in a 96-well plate for 24, 48, and 72 h.

### 2.7. Preclinical *In Vivo* Evaluation of EUG and NCEUG

#### 2.7.1. TPA-Induced Acute Ear Edema

Mice in groups (*n* = 8) were treated on their right ear with EUG or NCEUG (0.125; 0.25 and 0.5 mg/ear, 10 *μ*L), vehicle (1% acetone or NCB), or dexamethasone (0.05 mg/ear) prior to topical application of TPA. Ear thickness was recorded before and four hours after administration of TPA making use of a digital caliper (100.174B/Digimess®) [38]. Ear punch biopsies (5 mm) were collected for the determination of myeloperoxidase [39].

#### 2.7.2. Mouse Ear Edema Induced by Multiple Topical Applications of TPA

Chronic inflammation was induced by topical application of 20 *μ*L of TPA (2.5 *μ*g/ear) to the left ear of mice of each mouse with a micropipette on alternate days. The test compounds were dissolved in acetone and applied topically as the same concentrations of acute edema assay twice daily for four days, in the morning immediately after TPA application and 6 h later [38]. Dexamethasone was used as the reference drug (0.5 mg/ear). Ear thickness was recorded before and seven hours after administration of TPA making use of a digital caliper (100.174B/Digimess®).

#### 2.7.3. Measurement of IL-6 and Neutrophil KC (CXCL 1) Levels

Swiss mice had the left ear sample removed on day 4 for the analysis of cytokines. The samples were stored at −70°C until required for the assay. The collected tissue was homogenized and processed [40]. The concentrations of *IL*-*6* and *neutrophil KC* were determined using an enzyme-linked immunosorbent assay (ELISA) [41]. Briefly, microtiter plates were coated with an antibody against mouse *IL*-*6* and *neutrophil KC* (4 *μ*g/mL, DuoSet ELISA Development kit, R&D Systems) overnight at 4°C. After blocking the plates, the sample and standard were added at various dilutions in duplicate and incubated at 4°C for 2 h. The plates were washed three times with buffer. After washing the plates, biotinylated goat anti-mouse (diluted 1 : 1000 with assay buffer 1% BSA, R&D Systems, USA) was added to the wells. After a further incubation at room temperature for 2 h, the plates were washed, and 100 *μ*L of streptavidin-HRP diluted 1 : 200 was added. To the plate, 100 *μ*L of substrate solution (1 : 1 mixture of H_2_O_2_ and tetramethylbenzidine; R&D Systems, USA) was added, and the plate was incubated in the dark at room temperature for 20 min. The enzyme reaction was stopped with H_2_SO_4_2 N, and the absorbance was measured at 450 nm. The results are expressed as pg/g of tissue and reported as mean ± SEM.

### 2.8. Statistical Analysis

Statistical analyses were performed using GraphPad Prism 5.0 (USA), and the results are expressed as mean ± SD (formulations) or mean ± SEM (*in vitro* and *in vivo* tests). The comparison of means was performed using analysis of variance (ANOVA) followed by Tukey's test. Differences were considered statistically significant when *p* < 0.05.

## 3. Results and Discussion

### 3.1. EUG Is Not Cytotoxic and Did Not Alter Cell Membrane Stability

**Figure 1:**
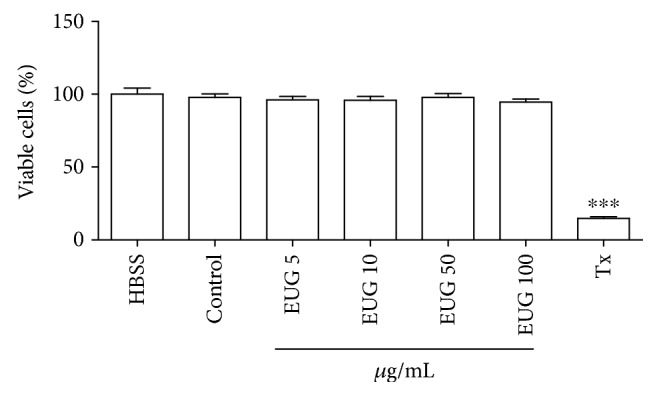
Evaluation of EUG toxicity on MTT test in human neutrophils. Data from three to eight samples. The control group consists of cells treated with vehicle (DMSO 1%). Triton X-100 (Tx, 0.02%) was used as cytotoxic standard. ^∗^vs. HBSS: nontreated cells. *p* < 0.05 (ANOVA and Tukey as the post hoc test).

In order to verify the possible cytotoxicity of EUG, we investigated its effects on cellular metabolism with the MTT test and cell membrane integrity in human neutrophils. As presented in Figure 1, the addition of EUG (5–100 *μ*g/mL) to neutrophils did not cause a significant reduction on cell viability (96.03 ± 2.52, 95.87 ± 2.63, 97.76 ± 2.64, and 94.59 ± 2.13%, respectively) measured by the MTT test after 30 minutes of incubation when compared to the control group (DMSO 1%: 97.86 ± 2.34%). In this assay, it was observed that EUG unlike Triton X-100 (standard, cytotoxic) did not interfere cell viability in relation to the groups control (vehicle) and RPMI (nontreated cells), suggesting the absence of toxicity on cellular metabolism, particularly related to the activity of the mitochondrial succinate dehydrogenase enzyme.

**Figure 2:**
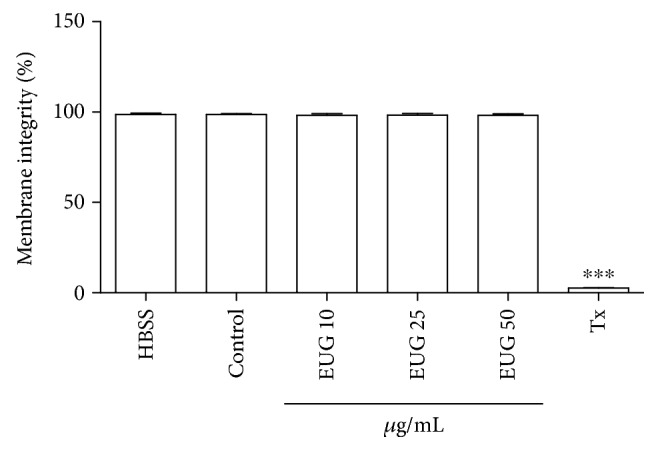
Effect of EUG on the percentage of measurement of membrane integrity of human neutrophils as monitored by flow cytometry using the sensitive fluorochrome propidium iodide. Neutrophils were cultured for 30 min in the presence of EUG at various concentrations (10–50 *μ*g/mL). Values are presented as means ± SEM of 3 separated experiments in triplicate and by analysis of 10,000 events. The control group consists of cells treated with vehicle (DMSO 1%). ^∗^vs. HBSS: nontreated cells. *p* < 0.001 (ANOVA and Tukey as the *post hoc* test).

Some assays may be used to investigate the possible effect of drugs on the pattern of cell death including the detection of cell morphological changes by light microscopy, electron microscopy, or flow cytometry [42]. Following the safety evaluation studies of EUG, the flow cytometry methodology was used, since the technique is a tool that provides in a quick way the objective of the determination of biological parameters such as cell size, cell type, DNA content, and enzymatic function. In the determination of membrane integrity, the dye propidium iodide (PI) was used, which has the capacity to bind to the DNA of the cell, allowing the identification of cellular viability and fragmentation of the nuclear material. PI penetrates only cells that have membrane changes, emitting high red fluorescence when excited at 488 nm [31]. Thus, the greater the fluorescence emitted by the cell, the greater the change in permeability of the plasma membrane that can culminate in cell death. The membrane integrity assessment in human neutrophils revealed that the incubation with EUG (10, 25, and 50 *μ*g/mL) within 30 minutes did not cause membrane disruption (98.21 ± 0.85, 98.28 ± 0.88, and 98.15 ± 0.77%, respectively) compared to the control group (98.50 ± 0.60) (Figure 2). Studies with flow cytometry showed that treatment with EUG did not promote a significant decrease in the number of cells when compared to the control group. A study by Thompson et al. [43] to investigate the effect of EUG on human neutrophils showed that incubation of the drug at the maximum concentration of 1 mM did not promote significant cytotoxicity. Both results suggest absence of toxicity on the human neutrophil.

### 3.2. EUG Prevents Human Neutrophil Degranulation

**Figure 3:**
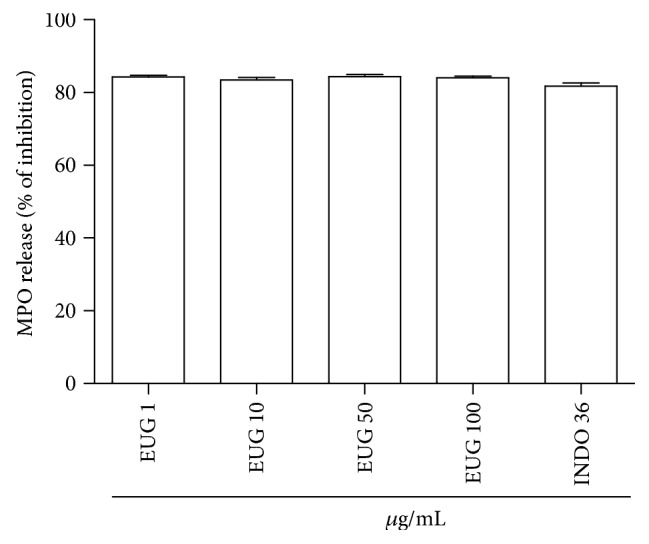
Effects of eugenol (EUG) on the release of human neutrophil myeloperoxidase (MPO) stimulated by phorbol myristate acetate (PMA). Freshly isolated cells (2.5 × 10^6^) were preincubated with indicated concentrations of EUG prior to the addition of PMA (0.1 *μ*g/mL). Indomethacin (36 *μ*g/mL) was used as positive control. Data are expressed as percentages of inhibition by EUG on the release of MPO. Numbers represent mean ± SEM. Data from three to eight samples.

The effect of EUG on neutrophil degranulation was investigated by measurement of myeloperoxidase release after the exposure of the cells to PMA (Figure 3). EUG (1–100 *μ*g/mL) and indomethacin (36 *μ*g/mL), a nonselective inhibitor of cyclooxygenase, inhibited the degranulation process of human neutrophil induced by PMA. EUG inhibited the PMA-stimulated neutrophil degranulation by 84%.

In the present study, we show that EUG inhibits proinflammatory mechanisms of human neutrophils, an effect that can contribute for the control of several inflammatory diseases such as dermatitis [44]. The treatment of human neutrophils with EUG significantly decreases the MPO amount released by the cells induced by PMA which is a receptor-independent stimulant which enters the cell and directly activates protein kinase C leading to the assembly and activation of NADPH oxidase [45]. MPO is a key constituent of the neutrophil granules that catalyzes the formation of hypochlorous acid, a compound with potent oxidant and microbicidal effects [46]. MPO not only serves as an index of neutrophil recruitment and activation but also displays cytokine-like properties that can serve to modulate the activation state of leukocytes in the inflammatory process [47]. In the present study, EUG strongly inhibited MPO release.

### 3.3. Treatment with EUG Inhibits Neutrophil ROS Generation

**Figure 4:**
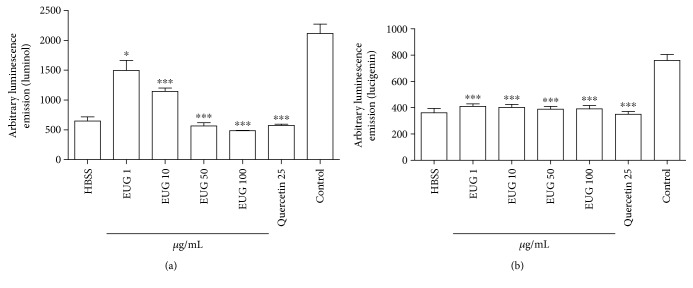
Evaluation of antioxidant activity of EUG in human neutrophils by chemiluminescence (CL). The inhibitory effect of EUG in human neutrophil oxidative metabolism was assessed by luminol (LumCL) (a) or lucigenin (LucCL) (b). Data from three to eight samples. ^∗^vs. control (DMSO), *p* < 0.05, and ^∗∗∗^vs. control (DMSO), *p* < 0.001 (ANOVA and Tukey as the *post hoc* test).

The inhibitory effect of EUG on the superoxide anion and total ROS generation by PMA-stimulated human neutrophil was assessed by the lucigenin- (LucCL-) and luminol- (LumCL-) enhanced chemiluminescence assays, respectively (Figure 4). EUG at 100 *μ*g/mL inhibited significantly (*p* < 0.05) the PMA-stimulated human neutrophils LumCL (77.16 ± 0.34%) and LucCL (51.40 ± 2.1%) (Figures 4(a) and 4(b), respectively). The reference compound quercetin at 25 *μ*g/mL inhibited LucCL and LumCL by 56.80% and 72.09%, respectively.

Oxidative stress is defined as an accumulation of reactive oxygen and nitrogen species that damage the structure of biomolecules such as DNA, lipids, carbohydrates, and proteins, as well as other cellular components, and can initiate inflammatory responses through the activation of redox-sensitive systems [48]. Furthermore, lipid degradation of the membrane (lipid peroxidation) induced by ROS can produce proinflammatory bioactive molecules that affect monocyte and neutrophil functions, which have an important role in several chronic inflammatory diseases [8, 49] such as dermatitis [50–52]. Thus, we investigated whether EUG had a modulatory effect in ROS production induced by PMA in human neutrophils measured by the luminol or lucigenin-enhanced chemiluminescence (CL) technique. It was observed that EUG reduced the ROS production in both luminol- or lucigenin-dependent chemiluminescence assays. The luminol probe can detect several intra- and extracellular reactive species, having higher sensitivity for the MPO-H_2_O_2_-HOCl system, while the lucigenin probe detects mainly production by neutrophils. The effect of EUG is relevant because excessive generation of oxidants by the superoxide anion and MPO-H_2_O_2_-HOCl system has been linked to tissue damage in many chronic inflammatory diseases, such as dermatitis [6]. The results obtained with EUG in this work corroborate previous studies demonstrating its antioxidant activity in PMA-stimulated neutrophils [43, 53] in which high concentrations of EUG (164.20 *μ*g/mL) have inhibitory activity of superoxide anion formation.

### 3.4. Physicochemical Characterization and Stability of Eugenol-Loaded Polymeric Nanocapsules

**Figure 5:**
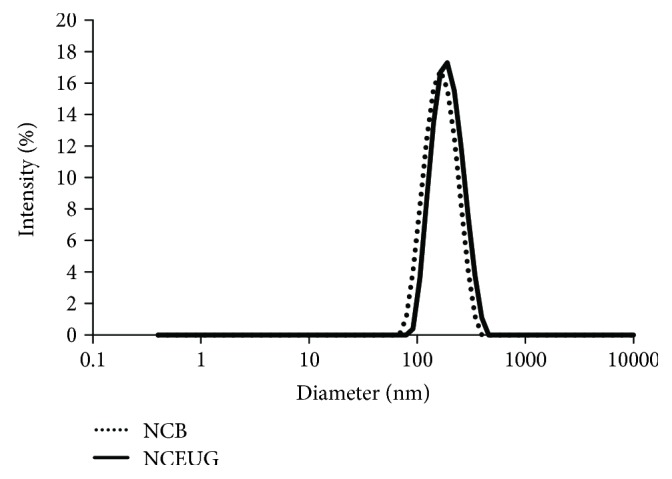
Size distribution by photon correlation spectroscopy of the eugenol-loaded nanocapsules (NCEUG) and the blank formulation (NCB).

The formulations presented unimodal size distribution (NCB: 146.1 ± 2.1 nm and NCEUG: 154.5 ± 1.4 nm) as shown in Figure 5. The polydispersity index indicated narrow distribution (PDI < 0.25). Zeta potential values, which reflect the surface charge of the nanoparticles, were −21.6 ± 1.3 mV and −13.2 ± 2.0 mV for NCEUG and NCB, respectively. The pH of the formulations were 3.4 (NCEUG) to 4.3 (NCB). The EUG content was 8.4 ± 0.5 mg/mL and the encapsulation efficiency 90%. Along 2 months of storage, no macroscopic changes such as creaming, sedimentation, or flocculation were observed. In addition, no changes in the size, PDI, zeta potential, pH, and EUG content were also verified (Table 1).

### 3.5. EUG Reduces Human Keratinocyte Viability, but Nanoencapsulation Reduces Its Cytotoxicity

**Figure 6:**
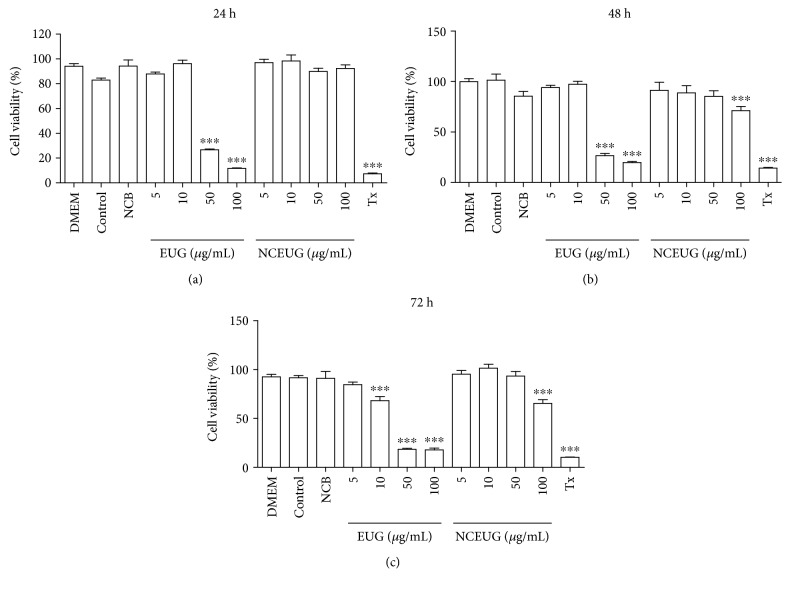
Evaluation of EUG and NCEUG toxicity on MTT test for 24 hours, 48 hours, and 72 hours in human neutrophils. Data from two to eight samples. The control group consists of cells treated with vehicle (DMSO 1%). ^∗^vs. DMEM: untreated cells. *p* < 0.05 (ANOVA and Tukey as the *post hoc* test).

As shown in Figure 6(a), it can be seen that the addition of EUG to human keratinocytes (HaCaT) caused a significant reduction in cell viability measured by the MTT test at concentrations of 50 and 100 *μ*g/mL (11.93 ± 1.18 and 26.69 ± 0.70, respectively) after 24 h of incubation when compared to the control group (DMSO 1%: 82.98 ± 1.53%). On incubation of 48 (Figure 6(b)) and 72 h (Figure 6(c)), EUG promoted reduction in cell viability at concentrations of 50 and 100 *μ*g/mL (26.50 ± 2.19 and 19.70 ± 1.11%) when compared to the control group (DMSO 1%—48 h: 101.5 ± 5.93%; 72 h: 91.51 ± 2.28%). Some studies of EUG and isoeugenol cytotoxicity demonstrated that these molecules promote growth suppression in keratinocytes (HaCaT) and that the effects may be mediated through interactions with the aryl hydrocarbon receptor (AHR) [54]. In a later study, Kalmes and Blömeke [55] demonstrated that the effects of EUG and isoeugenol were determined on AHR intracellular localization, expression of the target gene AHR, regulation of cell cycle-dependent AHR, and proliferation of HaCaT cells. Both compounds produced a rapid and marked translocation to the nucleus AHR, inducing the expression of target genes AHR as cytochrome P450 1A1 (CYP1A1) and repressor (AHR AhRR), and inhibited proliferation of the HaCaT cells, showing that antiproliferative properties of EUG and isoeugenol are mediated through AHR in HaCaT cells. Despite these results, EUG still possesses notorious antioxidant and anti-inflammatory effects, so that we used nanocarriers as a strategy to overcome both its cytotoxicity on keratinocytes and its volatility, which is a problem regarding the topical application. When incubated with NCEUG (5, 10, 50, and 100 *μ*g/mL), the viability of the keratinocytes was not reduced (87.52 ± 3.14, 96.94 ± 2.75, 98.33 ± 4 29; 89.84 ± 2.63, and 92.32 ± 2.93%, respectively) compared to the blank formulation (NCB, 94.20 ± 4.95%) (Figure 6(a)). However, the incubation of keratinocytes with NCEUG changed the viability of the cells only at a concentration of 100 *μ*g/mL (48 h: 71.14 ± 4.20; 72 h: 65.38 ± 3.84%) when compared to NCB (48 h: 85.53 ± 4.72%; 72 h: 90.89 ± 7.27%) (Figures 6(b) and 6(c)). The improvement of the biological responses after nanoencapsulation has been reported [56], and the successfulness of NP-based delivery has been associated with their nanorange size and excellent biopharmaceutical properties, such as high entrapment efficiency, controlled release rates, and insignificant enzymatic degradation. So, NCEUG seemed to be a promising formulation for the treatment of ICD and it was submitted to *in vivo* experiments.

### 3.6. Nanoencapsulation Improves the *In Vivo* Anti-inflammatory Properties of EUG

**Figure 7:**
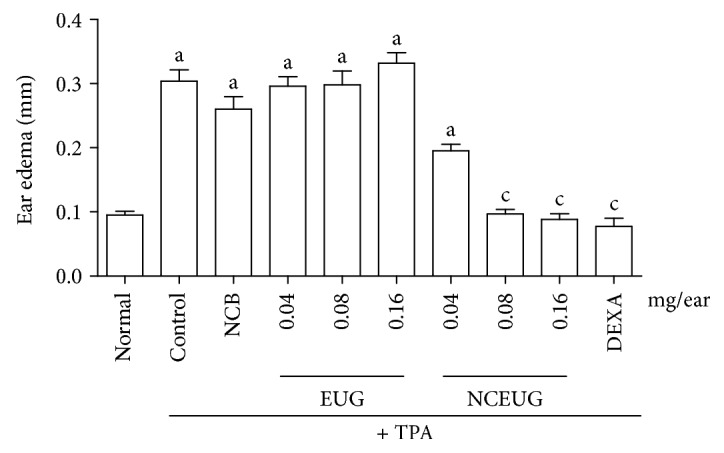
Effects from EUG and NCEUG on TPA-induced acute edema in mice. Swiss mice (25 to 30 g) were treated topically with EUG or NCEUG (0.04, 0.08, and 0.16 mg/ear), NCB (blank formulations), dexamethasone (DEXA; 0.05 mg/ear), or acetone (vehicle control) with topical application of TPA (2.5 *μ*g/ear) on the surface of the left ear of mice. Values represent mean ± SEM from 8 animals per group. a vs. the normal group; b vs. control; c vs. NCB group; *p* < 0.05 (ANOVA and Tukey's test).

Considering the possibility that the absence of efficacy of EUG could be related to its irritant effect on skin [57], a nanosystem using EUG as active principle was developed.  Figure 7 presents the antiedematogenic effect of the topical administration of EUG and NCEUG (0.04, 0.08, and 016 mg/ear) on TPA-induced ear edema in mice. Pretreatment of animals with EUG at doses of 0.04, 0.08, and 0.16 mg/ear did not promote reduction in ear edema thickness (0.29 ± 0.01, 0.29 ± 0.02, and 0.33 ± 0.01, respectively), when compared to the control group (0.32 ± 0.03), corresponding to inhibitions of 5.20%, 7.81%, and 7.50%. Dexamethasone (0.05 mg/ear), used as the standard drug, significantly inhibited the thickness (mm) of ear edema with inhibition of 85.59%. However, incorporation of EUG in nanocapsule suspensions (NCEUG; 0.08 and 0.16 mg/ear) significantly reduced TPA-induced ear edema (mm) in mice (0.10 ± 0.009, 0.08 ± 0.008), with inhibitions of 30.29, 61.61, and 68.82%, when compared to the control group (NCB: 0.22 ± 0.022). These results corroborated with previous studies which showed that nanocarriers (nanoemulsion, nanocapsules, and nanospheres) are alternatives to topical administration showing a controlled drug release and improving the efficacy in the treatment of contact dermatitis. It was demonstrated that polymeric nanocapsules did not produce contact sensitization in mice stimulated by oxazolone [58]. Also, it was reported that nanocapsules containing clobetasol propionate, a corticosteroid used for treatment of skin disorders, in a model of contact dermatitis after topical administration in rats led to a better control of the drug release and provided better *in vivo* dermatological efficacy [59]. So, possibly this anti-inflammatory activity instead of an irritant effect on the skin observed in this study is related to the drug controlling on skin permeation.

**Figure 8:**
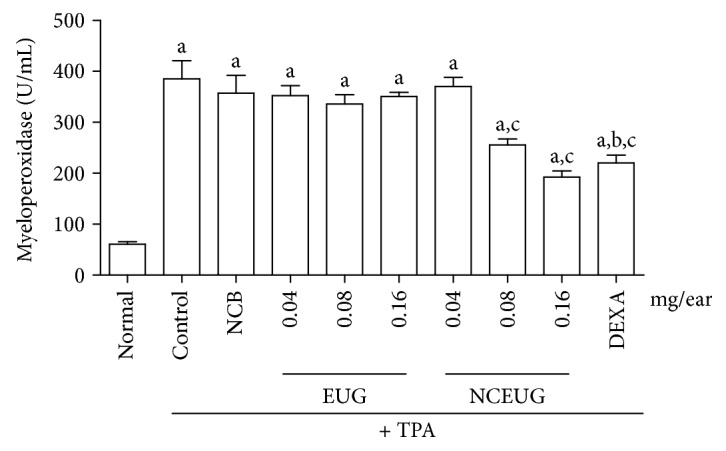
Effect of EUG on myeloperoxidase activity induced by TPA in mice. Swiss mice (25 to 30 g) were treated topically with EUG or NCEUG (0.04, 0.08, and 0.16 mg/ear), dexamethasone (DEXA; 0.05 mg/ear), or acetone (vehicle control) with topical application of TPA (2.5 *μ*g/ear) on the surface of the left ear of mice. Values represent mean ± SEM the amount of MPO in U/mL. 8 animals were used per group. a vs the normal group; b vs control group; c vs NCB; *p* < 0.05 (ANOVA and Tukey's test).

The topical administration of EUG and NCEUG on the measurement of myeloperoxidase (MPO) in tissue of mice submitted to ear contact dermatitis induction with TPA (Figure 8) indicates that the treatment of animals with EUG at concentrations of 0.04, 0.08, and 0.16 mg/ear were not able to reduce leukocyte infiltration expressed through the MPO levels (352.10 ± 19.84 and 336.0 ± 17.97350 ± 8.57 U/mL) corresponding to inhibitions of 18.75, 15.93, and 10.40%, respectively, compared to the control group (386.20 ± 13.52 U/mL). Dexamethasone (0.05 mg/ear), an anti-inflammatory drug standard, promoted reduction of MPO by 129.50 ± 5.54 U/mL (66.40% inhibition). NCEUG at concentrations of 0.08 and 0.16 mg/ear reduced significantly (*p* < 0.05) the amount of MPO (255.70 ± 11.76 U/mL and 192.10 ± 12.33 U/mL), with inhibitions of 32.01 and 50.33%, respectively, compared to the control group (NCB: 320.70 ± 29.96 U/mL, inhibition of 16.14%).

**Figure 9:**
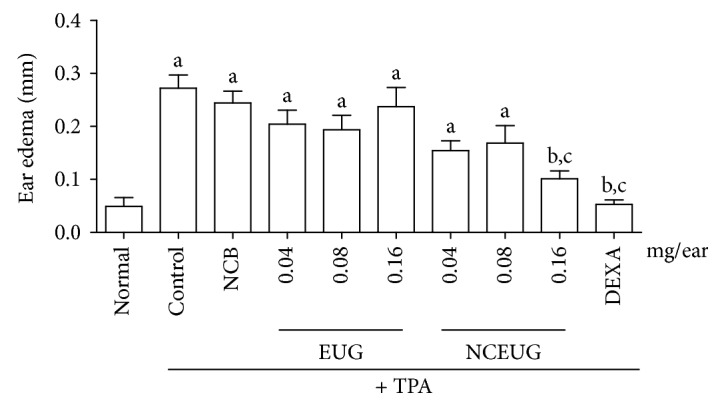
Antiedematogenic activity from EUG and NCEUG induced by TPA in mice. Swiss mice (25 to 30 g) were treated topically with EUG or NCEUG (0.04, 0.08, and 0.16 mg/ear), NCB (blank formulations), dexamethasone (DEXA; 0.05 mg/ear), or acetone (vehicle control) with topical application of TPA (2.5 *μ*g/ear) on the surface of the left ear of mice. Values represent mean ± SEM from 8 animals per group. a vs the normal group; b vs control; c vs NCB group; *p* < 0.05 (ANOVA and Tukey's test).

Figure 9 demonstrates the effect of multiple topical administrations of EUG and NCEUG (0.04, 0.08 and 0.16 mg/ear) in the ear edema induced by TPA in mice. Pretreatment of animals with EUG in all doses did not interfere significantly in the thickness (mm) of ear edema (0.20 ± 0.02, 0.24 ± 0.03, and 0.19 ± 0.02, respectively) when compared to the control group (0.22 ± 0.02), corresponding to inhibitions of 10%, 9%, and 13%. Dexamethasone (0.05 mg/ear, standard drug) inhibited significantly the ear thickness (mm) with an inhibition value of 77% (0.05 ± 0.008). Similar to acute ear edema, the incorporation of the nanocapsule suspension of EUG (NCEUG; 0.16 mg/ear) significantly reduced the ear edema (mm) induced by TPA in mice (0.10 ± 0.01) with inhibition of 54% when compared to the control group (NCB: 0.24 ± 0.02; 9% of inhibition) promoting effects similar to dexamethasone, an anti-inflammatory standard drug.

**Figure 10:**
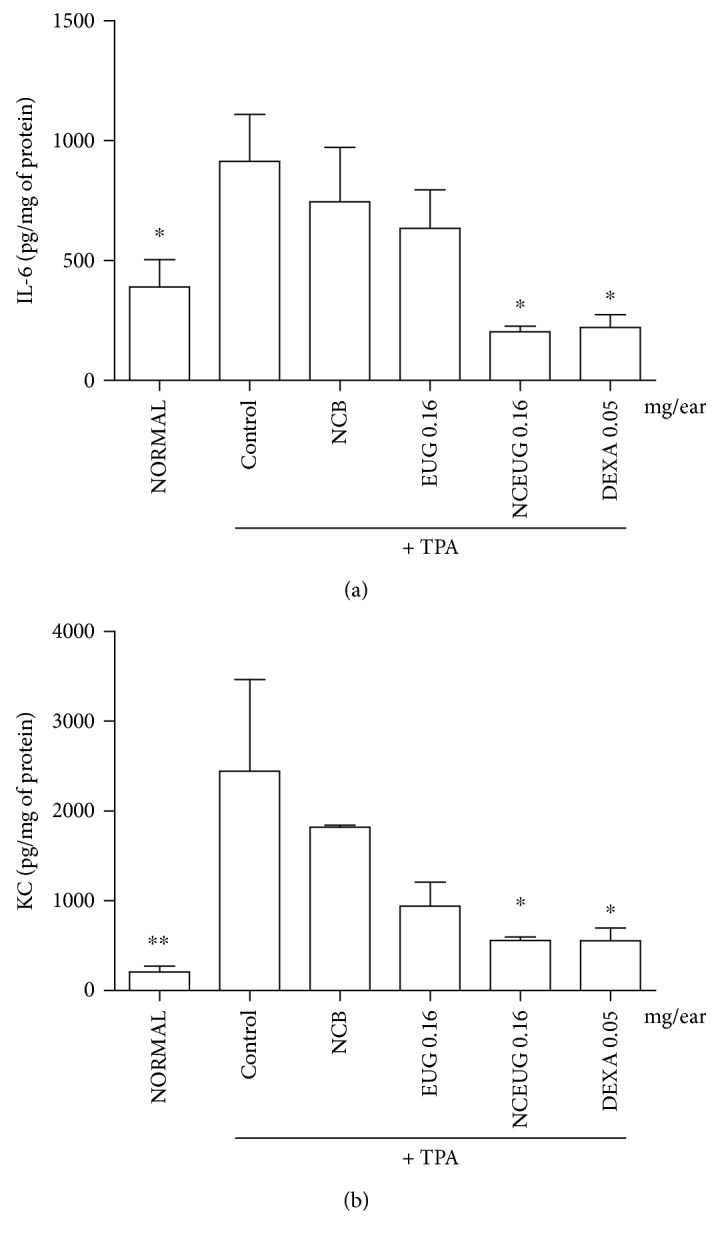
The effect of NCEUG from EUG and NCEUG on IL-6 (a) and KC (b) levels in ear tissue homogenates induced by TPA in mice. Swiss mice (25 to 30 g) were treated topically with EUG or NCEUG (0.04, 0.08, and 0.16 mg/ear), NCB (blank formulations), dexamethasone (DEXA; 0.05 mg/ear), or acetone (vehicle control) with topical application of TPA (2.5 *μ*g/ear) on the surface of the left ear of mice. Values represent mean ± SEM from 8 animals per group. ^∗^vs control group; *p* < 0.05 (ANOVA and Dunnett's test).

Repeated application of TPA to sensitized mice caused an increase in tissue levels of IL-6 and KC levels. The treatment with NCEUG (0.16 mg/ear) significantly suppressed the TPA-induced IL-6 and neutrophil KC levels, yielding an average inhibition of 77% for both mediators (Figure 10).

It was observed in the results that the treatment of the animals with NCEUG (0.08 and 0.16 mg/ear) caused a significant reduction in the ear swelling (mm) on TPA-induced mice when compared to the EUG group. These results corroborated with previous studies which showed that nanocarriers (nanoemulsion, nanocapsules, and nanospheres) are alternatives to topical administration showing a controlled drug release and improving the efficacy in the treatment of contact dermatitis. It was demonstrated that polymeric nanocapsules did not produce contact sensitization in mice stimulated by oxazolone [58]. Also, it was reported that in a model of contact dermatitis after topical administration in rat nanocapsules containing clobetasol propionate, a corticosteroid used for treatment of skin disorders, it led to a better control of the drug release and provided better *in vivo* dermatological efficacy [59]. So, it is possible that the nanoencapsulation of EUG controlling the drug permeation on the skin allowed terpene to show an anti-inflammatory activity instead of the irritant effect on the skin.

There are few studies evaluating the effects of EUG in the ear edema model in mice (commonly employed for research of inflammatory skin diseases such as dermatitis). Among them, one showed that EUG at concentrations of 0.2 and 0.5 mg/ear promotes significant reduction of edema induced by croton oil [60]. In disagreement, in the present study, EUG (0.04, 0.08, and 0.16 mg/ear) did not show anti-inflammatory effects in both models of mouse edema ear (acute and multiple). This result may be related at least in part with the lower dose of EUG and the type of skin irritant; although croton oil contains 12-*o*-tetradecanoylphorbol-13-acetate (TPA) and other phorbol esters as main irritant agents, our study used the pure skin agent where, in skin and keratinocytes, TPA had a biphasic influence, stimulating immature basal cells to proliferate while accelerating differentiation in committed cells [60].

In addition, the acute assay of irritant contact dermatitis induced by TPA in mice using EUG showed that this terpene has no antiedematogenic action, but its incorporation into the polymeric nanocapsules reduced significantly the ear thickness after induction of edema with TPA similar to dexamethasone (standard drug). At least part of the anti-inflammatory activity of NCEUG is related to its ability to reduce the levels of MPO, IL-6, and KC (CXCL 1), markers of cell accumulation into the inflammatory focus. Polymeric nanoparticles (NP) have been applied to immunomodulatory therapies to enhance their efficacy and reduce potential side effects on the stratum corneum [61, 62] and are well recognized as an advanced noninvasive technique to facilitate delivery of therapeutics into the skin [63]. These properties can be engineered to make them suitable for specific biomedical applications. Some studies showed that the hydrocortisone-loaded polymeric NPs were more efficient in alleviating the signs and symptoms of dermatosis in mice compared to a hydrocortisone cream of equivalent and higher concentrations [64, 65].

## 4. Conclusion

The present study showed that EUG, a bioactive terpene present in essential oils of medicinal plants, inhibited the ROS production in human neutrophil, but it was toxic in human keratinocyte and did not interfere with ear edema induced by TPA. However, the nanoencapsulation of EUG (NCEUG) prevented its cytotoxicity in keratinocytes and reduced ear thickness of mice reducing the MPO activity and the concentrations of IL-6 and KC (CXCL 1). Together, these results showed that NCEUG promoted a reduction in cytotoxicity of EUG and improved its anti-inflammatory effect. However, further studies are necessary to elucidate the antioxidant and anti-inflammatory mechanisms of action in order to determine their potential in the treatment of topic diseases, such as contact dermatitis.

## Figures and Tables

**Table 1:** Stability of the eugenol-loaded nanocapsules stored at room temperature for 2 months.

Day	Diameter (nm)	PDI	Zeta potential (mV)	pH	Drug content (%)
1	154 ± 1	0.03 ± 0.01	−20.9 ± 0.4	4.1 ± 0.3	83.4 ± 0.5
30	159 ± 1	0.04 ± 0.01	−23.8 ± 0.4	4.2 ± 0.1	83.3 ± 0.4
60	158 ± 4	0.04 ± 0.02	−18.8 ± 0.1	4.3 ± 0.1	83.3 ± 0.3

The values represent mean ± SD (*n* = 3).

## Data Availability

All other data arising from this study are contained within the manuscript file.
